# Classification performance assessment for imbalanced multiclass data

**DOI:** 10.1038/s41598-024-61365-z

**Published:** 2024-05-10

**Authors:** Jesús S. Aguilar-Ruiz, Marcin Michalak

**Affiliations:** 1 School of Engineering, Pablo de Olavide University, 41013 Seville, Spain; 2 Department of Computer Networks and Systems, Silesian University of Technology, ul. Akademicka 16, 44-100 Gliwice, Poland

**Keywords:** Computer science, Information technology

## Abstract

The evaluation of diagnostic systems is pivotal for ensuring the deployment of high-quality solutions, especially given the pronounced context-sensitivity of certain systems, particularly in fields such as biomedicine. Of notable importance are predictive models where the target variable can encompass multiple values (multiclass), especially when these classes exhibit substantial frequency disparities (imbalance). In this study, we introduce the Imbalanced Multiclass Classification Performance (IMCP) curve, specifically designed for multiclass datasets (unlike the ROC curve), and characterized by its resilience to class distribution variations (in contrast to accuracy or F_β_-score). Moreover, the IMCP curve facilitates individual performance assessment for each class within the diagnostic system, shedding light on the confidence associated with each prediction—an aspect of particular significance in medical diagnosis. Empirical experiments conducted with real-world data in a multiclass context (involving 35 types of tumors) featuring a high level of imbalance demonstrate that both the IMCP curve and the area under the IMCP curve serve as excellent indicators of classification quality.

## Introduction

Measuring the accuracy of diagnostic systems^1^ is an old challenge. In general, a suitable measure of performance must be independent of event frequencies and decision criteria. In machine learning, the classification task deals with assigning a class label to a given observation. To do that, a classification model has been previously learned with training data. On many occasions, the distribution of samples in known classes is skewed, i.e. the proportion of examples that belong to each class is unequal. Class distribution refers to the relative frequency of examples of each class in the dataset, and when these frequencies are not comparable, it is named class imbalance. In the recent scientific literature, we find numerous examples of binary class imbalance (breast cancer prediction^2,3^; cognitive impairment in Parkinson’s disease^4^) and multi-class imbalance (31 cancer type classification^5^; identification of Parkinson’s disease subtypes^6^; microbiome-based cancer diagnostics^7^; prostate cancer detection^8^; dementia subtype prediction^9^; Gleason grading of prostate cancer^10^; severity of COVID-19 pneumonia^11^; faecal microbiome-based multi-class disease diagnosis^12^).

**Figure 1:**
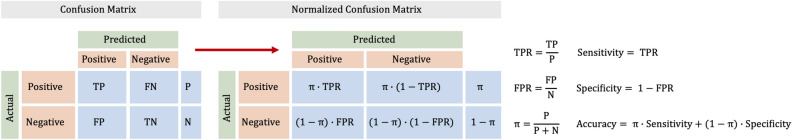
Confusion matrix as a function of true positive rate (TPR), false positive rate (FPR) and the positive class ratio ($\pi$).

Class imbalance is another challenge for classification assessment, as most of the evaluation metrics are sensitive to data distribution, i.e. to class sizes. Let *D* be a dataset with *n* samples and two classes (Positive and Negative). The confusion matrix is represented by a $2\times 2$ matrix with True Positives (TP), False Negatives (FN), False Positives (FP), and True Negatives (TN), as illustrated in Fig. 1 (left: Confusion Matrix). The sum of all positives (negatives) is P (N). The matrix can be normalized by dividing by the number of samples $n=\text {P}+\text {N}$, and all the values can be expressed in terms of the True Positive Rate (TPR), the False Positive Rate (FPR) and the proportion of positives ($\pi$), as shown in Fig. 1 (right: Normalized Confusion Matrix). Thus, the sum of all positives is $n\pi$, and of all negatives is $n(1-\pi )$. Both sensitivity (TPR) and specificity ($1-\text {FPR}$) are not influenced by class distribution ($\pi$). However, the accuracy measure contains $\pi$ in its mathematical expression, i.e. it is highly dependent on class bias, so it is exclusively valid for balanced datasets. Despite the uselessness of accuracy for imbalanced data^13^, it is still the most popular evaluation measure for classification. Precision, another well–known measure that calculates the ratio between true positive predictions and all the positive predictions, is also sensitive to class skew (expressed in function of the normalized confusion matrix, it also includes $\pi$), and thus F_β_–score^14,15^, which combines precision and sensitivity, also has the same drawback. Therefore, any measure without the presence of $\pi$, like the geometric mean between sensitivity and specificity (G-measure)^16^, is not influenced by the imbalance between the classes.

In order to mitigate imbalance in datasets two main techniques are approached: under-sampling and over-sampling. Under-sampling^17–20^ means reducing the size of the data to equal the size of the minority (lowest frequency) class, which is never recommended as it loses information for training the classifier. Instead, over-sampling attempts to match the size of all classes to that of the most frequent class, so it does not lose information but it has to generate new samples for the underrepresented classes. This generation can be natural (copies of existing samples) or synthetic (completely new samples from the existing ones). The application of any resampling technique would likely lead to different classification performance, trying to improve the performance before resampling. However, resampling techniques are only focused on the training phase, when the classification model learns. During the testing phase the imbalance will still be present, so the performance metrics must be able to manage it properly. For instance, it would not be correct to apply resampling before cross-validation; the right method would be applying cross-validation and for each training set apply resampling, keeping the test set with the original class distribution. Somehow, modifying the class distribution for the test phase would be equivalent to altering the reality that the classifier is going to face and, therefore, the performance results would be adulterated.

Among the best known sampling techniques, SMOTE^21^ has gained popularity. It generates new minority class objects as weighted average of closest neighbors (from the same minority class), and the results during the training phase support its good acceptance in the scientific community^22–26^. Nevertheless, the performance of the classifier—even with better quality learning due to training balance—still depends on the imbalance of the test set. Consequently, classification performance measures should not be sensitive to class bias.

The ROC curve^27–29^ represents the relation between the FPR and the TPR, and illustrates the diagnostic ability of a binary classifier as its discrimination threshold is varied from the confusion matrix. The curve shows a solid idea of the behavior of a classifier and allows to compare several classifiers in one image for the same dataset. Most importantly, the ROC curve, unlike the accuracy or F-measure, only depends on the FPR and TPR, which are not class biased. Given its usefulness, the ROC curve has been used in many works^30–34^, although it has an enormous limitation: it can only be used in binary contexts, i.e. two-class datasets. In case of multi-class datasets, the most common approach is to represent the class of interest (positive) against the others (negative), but it does not contribute to understanding the global performance of the classifier. In case of equal importance of classes, the smoothed solution named *macro-average* is to average all the ROC curves (one-versus-rest for each class), but this compromises its natural insensitivity to class skew. On the contrary, the *micro-average* approach takes into account the proportion of every class (aggregating the contributions of all classes), given more importance to larger classes and, therefore, assigning greater values when the largest class performs better. However when the imbalance is significant, both approaches lead to very different curves with refutable interpretations.

The ROC curve has been proven that is an excellent measure of classification performance^35,36^, as it conveys more information than many scoring metrics by visualizing the performance of the classifier by a curve instead of providing a single scalar value. However, the ROC curve also provides a comparable measure of performance: the area under the ROC curve (AU(ROC)). There exist approaches for the AU(ROC) in the context of multi-class classification, based on averaging pairwise comparison of classes^37^ or on the use of *K*-dimensional space to compute the volume of the ROC surface^38–41^, being *K* the number of class labels. However, none of these approaches provide a graphical representation.

In general, when the binary case (positive and negative) is extended to the multi-class case, the class imbalance becomes more prominent. Recently, the Multi-class Classification Performance (MCP) curve solved the problem of showing in a single curve the performance of multi-class datasets for any classifier^42^. However, the MCP curve is also sensitive to class bias. In this work, we introduce the Imbalanced Multi-class Classification Performance (IMCP) curve, as a graphical representation of the performance of the classifier, which is independent of the class distribution, and also the Area Under the IMCP curve (AU(IMCP)), as a scalar measure of prediction quality. Results show the usefulness of both the IMCP curve and the AU(IMCP), firstly with synthetic data to highlight the features of the IMCP curve when analyzing data with class imbalance, and secondly with real–world data to provide an example in which common performance metrics fail at determining the quality of classifiers.

## Rationale

**Figure 2:**
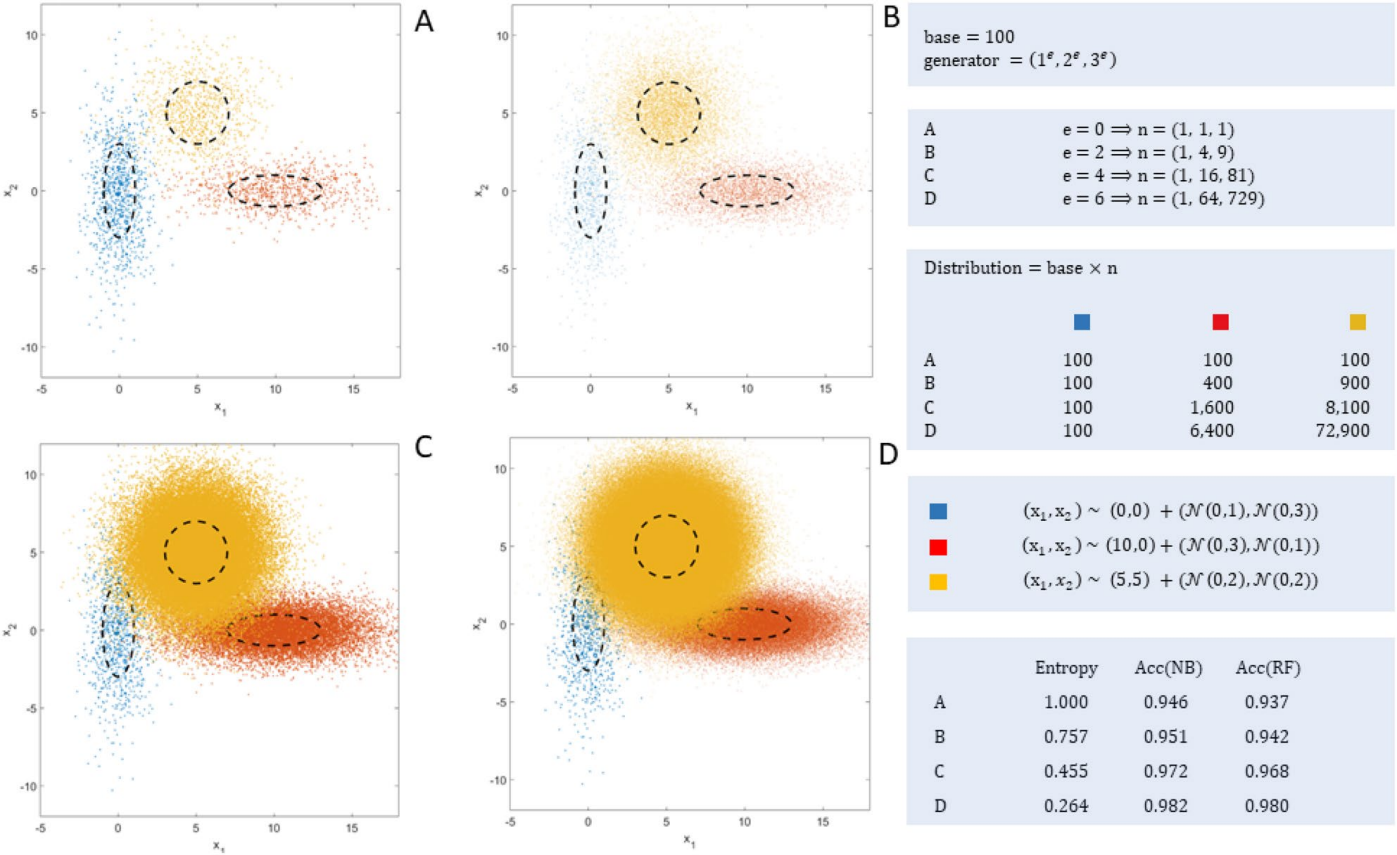
Samples with different distribution of classes.

The hypothesis is that a consistent assessment metric should calculate classification performance independently of the distribution of the *K* classes, so that overrepresented classes have no further impact on performance results. To show the impact of the degree of imbalance on classification performance, four synthetic 3-class datasets have been generated, using exactly the same generating functions for each dataset, but varying the distribution of classes exponentially. Figure 2 (left) shows the four datasets, denoted by the letters A, B, C, and D. The datasets have two variables—to simplify visualization on the X and Y axes—and three classes, denoted by the colors blue, red and yellow.

To provide different degrees of imbalance an exponential methodology supported by a *base* and a *generator* has been designed. The number of samples of each dataset is obtained by multiplying the *base* by the *generator*, which is based on the tuple (1, 2, 3) raised to an exponent *e* that controls the degree of imbalance. The base has been set to 100, and the exponent takes the values 0 (dataset A), 2 (dataset B), 4 (dataset C), and 6 (dataset D). Figure 2 (right) shows the distribution of the tuples for the values of the exponent. To generate the values of each variable a normal distribution has been used, $X, Y \sim \mathscr {N}(\mu ,\,\sigma ^{2})$, locating the centers at (0,0), (10,0) and (5,5), for blue, red and yellow classes, respectively, and slightly varying the deviations from 1 to 3 to make the shapes somewhat different (ellipses). This method is very reproducible and extensive to more classes, as there is only need to define the base and the exponent for setting the number of examples for each dataset.

The entropy of each dataset is a good indicator of the class imbalance, ranging from 1 (dataset A: zero imbalance) to 0.264 (dataset D: huge imbalance). The worst case is when $e = 6$, which provides an imbalance of 0.13% (blue), 8.06% (red), and 91.81% (yellow), visible in Fig. 2 (dataset D). Also, a stratified 10-fold cross-validation was used to compute the accuracy of two well–known techniques: Naïve-Bayes (NB) and Random Forests (RF), which have been chosen to facilitate replicability. These results are also shown in Fig. 2 (lower right corner), and as expected, the accuracy of both methods increases as the imbalance becomes greater (or entropy smaller). In short, there has been an unfair relative improvement in accuracy of 3.8% and 4.6% due to an imbalance for NB and RF, respectively, when transitioning from dataset A to D.

**Figure 3:**
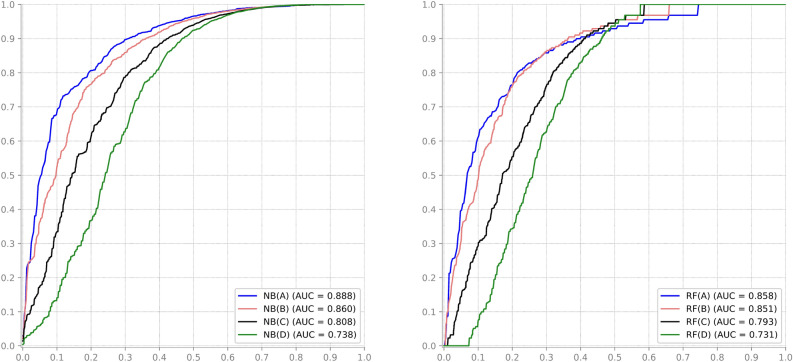
Evolution of the IMCP curve with increasing class imbalance for Naïve-Bayes (left) and Random Forests (right) classifiers, maintaining the data distributions. Class proportions of datasets A, B, C, and D are described in Fig. 2.

The impact of imbalance on classification performance is obvious when metrics sensitive to class bias are used. To avoid that negative impact, a novel method is introduced: the Imbalanced Multi-class Classification Performance (IMCP) curve. The IMCP curve is based on the MCP curve^42^, which was introduced to deal with multi-class datasets, unlike the ROC curve, which can only visualize the performance of binary (2-class) datasets. While the IMCP curve will be described in detail in the next section, Fig. 3 displays the results for the datasets outlined in Fig. 2. These results indicate that the increasing imbalance negatively impacts the classifier’s performance, in contrast to accuracy, which actually benefits from the heightened imbalance. In summary, as class imbalance increases, the results of the IMCP curve worsen for both NB and RF. This decline is also evident in the area under the IMCP curve, indicated at the lower left corner of each graph. This is in contrast to the accuracy depicted in Fig. 2, which exhibits remarkable sensitivity to class imbalance. In summary, classifiers that are trained with progressively higher degrees of class imbalance and then tested under the same imbalanced conditions tend to display a misleading increase in classification accuracy. However, when observing the IMCP curve and its corresponding AU(IMCP) under these identical conditions, a decreasing trend becomes apparent. This suggests that the latter metric more accurately captures the diminishing ability to correctly identify samples from the less frequent classes.

## Method

**Figure 4:**
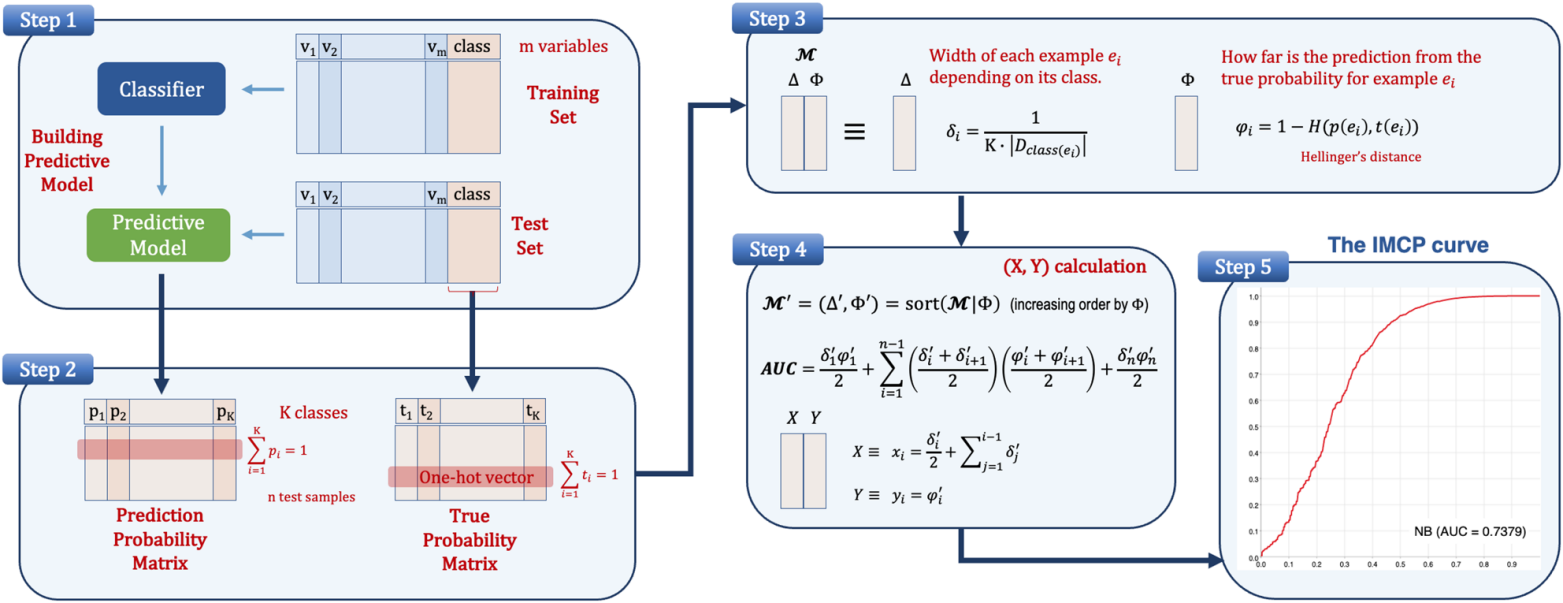
Description of the methodology for any classifier: from data to the IMCP curve.

For the sake of clarity, the method has been organized into five steps, as depicted in Fig. 4. Let $D=\{e_i \mid e=(\overline{x},y), \overline{x}\in \mathbb {X}^m, y \in \mathbb {Y}, i=1,2,\dots ,n \}$ be a dataset with *n* samples, which belong to a *m*-dimensional feature space and have a corresponding outcome *y* in the space $\mathbb {Y}$. When $\mid\mathbb{Y}\hspace{-0.1cm}\mid =2$ we say that it is a 2-class (binary) classification problem; otherwise, when $\mid \mathbb{Y}\hspace{-0.1cm}\mid >2$, we say that it is a multi–class classification problem. For simplicity, let us assume that $\mathbb {Y}=\{1,2,\dots ,K\}$ (set of class labels).

The first step is a common evaluation of classifiers by means of any validation technique. In this case, it is visualized a hold-out, but it should ideally be a stratified $\lambda$-fold cross-validation (commonly, $\lambda =10$), which would provide test results of the same size that the original dataset.

The second step provides two matrices: the prediction probability and the true probability matrices. The true probability of sample $e_i$, denoted by $t(e_i)$, can be encoded as a *K*-dimensional one-hot vector, in which all the values are 0 except for one 1 at the position *k*, that satisfies $y_i=k$ (following the *indicator function*, for all *k*, $t(e_i)_k=1_{y_i=k}$), being $k \in \mathbb {Y}$. The true probability matrix is in fact the binarization of the target variable (class). Consequently, the sum of all the values of the vector is 1. The prediction probability of sample $e_i$, denoted by $p(e_i)$, is also a *K*-dimensional vector, whose values are provided by the classifier as output probabilities of belonging to class $k \in \mathbb {Y}$. Most classification techniques (e.g. Naïve-Bayes, Decision Trees, Random Forests, Neural Networks, etc.) automatically provide the prediction probability vector for each test sample. This vector contains the probabilities that the test sample belongs to each class, so assuming there are *K* classes, the dimensionality is *K*, and the sum of all the probabilities is 1. From these two matrices that contain the prediction and true probabilities of each test sample, we could apply probability distribution distances in order to know how far is the prediction probability from the true probability.

The third step calculates two values for each test sample, located at columns $\Delta$ and $\Phi$ in matrix $\mathscr {M}$: $\Delta$ is associated with the X-axis and $\Phi$ with the Y-axis. Column $\Delta$ calculates the width $\delta _i$ associated to each test sample $e_i$ in the X-axis, that depends on the class distribution (examples belonging to overrepresented class will have smaller values). The sum of all $\delta _i$ is 1, but the sum of all $\delta _i$ associated with each class is the same, i.e., it is exactly $\frac{1}{K}$, being *K* the number of classes. In order to observe the quality of the classifier prediction, a distance function *d* between the two discrete probability distributions must be applied to each sample $e \in D$. Let *d* be a distance function between two distributions *t* and *p*, such as $d: \{0,1\}^K \times [0,1]^K \rightarrow [0,1]$. The Hellinger distance^43,44^ is a metric oriented to probability distributions, and has been chosen due to its interesting properties^42^. Let $P=[p_1, \ldots , p_K]$ and $Q=[q_1, \ldots , q_K]$ be two discrete probability distributions, the Hellinger distance is defined as:

1 $$\begin{aligned} H(P,Q)=\frac{1}{\sqrt{2}} \left( \sum _{j=1}^K \left( \sqrt{p_j}-\sqrt{q_j} \right) ^2\right) ^{\frac{1}{2}} \end{aligned}$$

Column $\Phi$ first calculates the Hellinger distance $H(p(e_i),t(e_i))$ between the prediction probability and the true probability of each sample $e_i$. If the distance between the true probability $t(e_i)$ and the prediction probability $p(e_i)$ of a sample $e_i$ is close to 0, then the classifier is making a good prediction for $e_i$; otherwise, when it is close to 1, the prediction is bad. Good or bad predictions are not necessarily correct and incorrect, respectively. The distance is a quality measure of the classification performance, so it would be possible to provide at the same time a not small distance associated with a correct prediction (e.g., for a 3-class problem, when t=[1,0,0] and p=[0.35,0.33,0.32], H(t,p)=0.639—very high value—and the prediction is correct). This fact provides interesting insights to address the uncertainty of classifiers, since even when they correctly assign the class the value of the highest probability might not be far from the others.

The distance value is very important because it informs about how far the prediction is from the true observation for each sample. The value $\varphi _i=1-H(p(e_i),t(e_i))$ can be interpreted as the probability that the classifier correctly assigns the observed class label to the test sample $e_i$. If $\varphi _i$ is zero, then the distance *H* between prediction and true probabilities is maximum (one). On the contrary, if $\varphi _i$ is one, then the distance *H* is zero, which means that prediction and true probabilities are equal, i.e., the prediction probability vector is a one–hot vector similar to the true probability vector.

The fourth step prepares the values of columns $\Delta$ and $\Phi$ to be visualized as X-axis and Y-axis, respectively. First, the matrix $\mathscr {M}$ is increasingly sorted according to column $\Phi$, providing $\Phi ^\prime$. Once obtained the matrix $\mathscr {M}^\prime =(\Delta ^\prime ,\Phi ^\prime )$, the AU(IMCP) can be easily calculated following the expression provided in Fig 4 (Step 4).

The new values in the $\Delta ^\prime$ column are then used to calculate the values for X-axis of the IMCP curve, while the values of Y-axis are the same as in $\Phi ^\prime$.

The fifth step draws the points of the (*X*, *Y*) matrix in the unit square, as well as visualizes the area under the IMCP curve. The first point ($x=0$) and the last point ($x=1$) retain the first and last values of the $\Phi ^\prime$ column, i.e. these will be the points $(0,\varphi _1)$ and $(1, \varphi _n)$.

**Figure 5:**
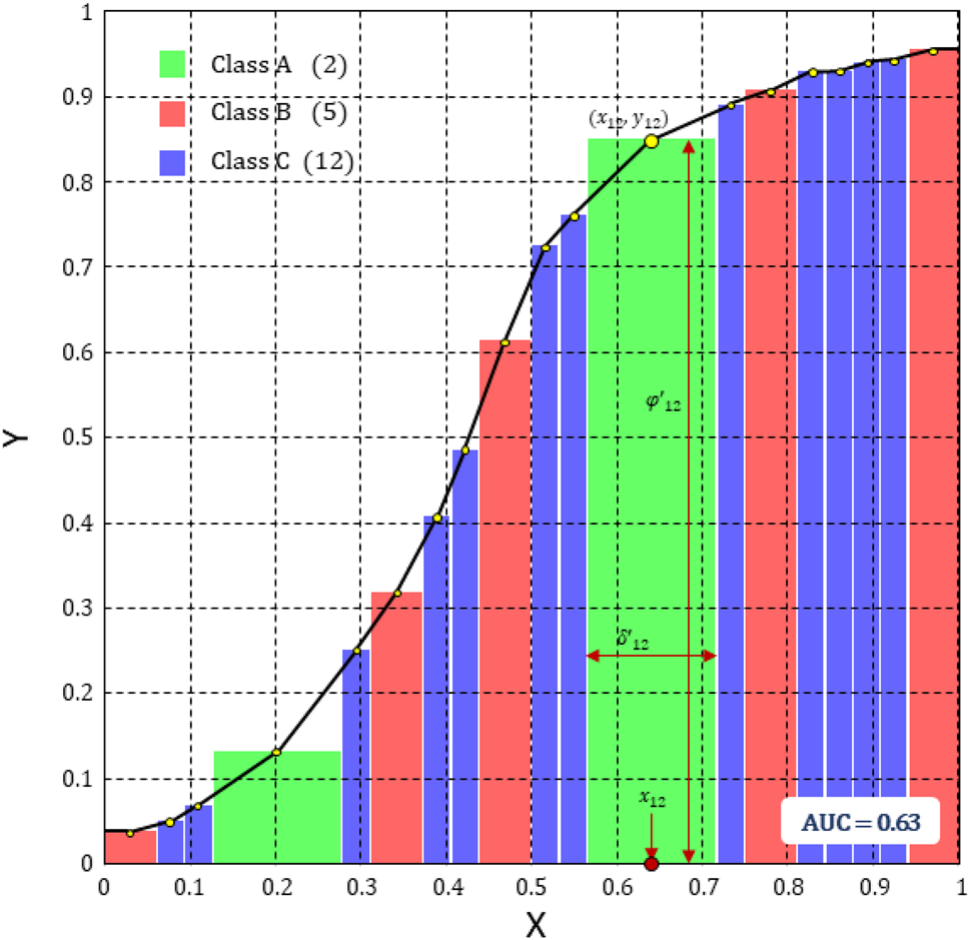
The IMCP curve for a simple example with three imbalanced classes (2 samples of class A, 5 of class B, and 12 of class C.

In order to highlight the influence of the class distribution in the dataset, Fig. 5 shows a synthetic example in which the importance of each class is balanced, while varying proportionally the importance of each sample. The role of $\delta ^\prime$, $\varphi ^\prime$, *x* and *y* in the method is illustrated in Fig. 5 (e.g., for position $i=12$ in (*X*, *Y*)).

The features of the IMCP curve provide interesting aspects related to the interpretability and explainability of predictive models:

- Upper bound: the ideal classifier should display the function $y=1$, that has $\text {AU(IMCP)}=1$, which means that for each test sample the distance between prediction probability and true probability is zero, i.e. the probability of assigning the true class is always one.
- Lower bound: the worst case is the function $y=0$, which has $\text {AU(IMCP)}=0$, which means that for each test sample the prediction probability assigned to the true class is zero.
- Correct classification threshold ($\eta$): the Hellinger distance can be expressed as a function of the symmetric Bhattacharyya coefficient (BC)^45^, where $\text {BC}(P,Q)=\sum _{j=1}^{K}\sqrt{p_j q_j}$ and $\text {H}(P,Q)=\sqrt{1-\text {BC}(P,Q)}$; if $\text {BC}>\sqrt{0.5}$ then the predicted class will be the true class. Therefore, if $\varphi >1-\eta =1-\sqrt{1-\sqrt{0.5}}=0.459$ then the test sample will be correctly satisfied (the opposite is not true).
- Incorrect classification threshold ($\theta$): the threshold for a correct prediction for a *K*-classification problem ($K>1$) is $\theta =\sqrt{1-\frac{1}{\sqrt{K}}}$. Therefore, if $\varphi <1-\theta$ then the test sample will not be correctly classified.
- Uncertainty: correct and incorrect classification can be defined in terms of the $\varphi$ values. However, there is an interval of uncertainty $[1-\theta , 1-\eta ]$ in which all the prediction probabilities for each class are below 0.5, so the difference between the best and the second best values could be very close to zero.

For the ROC curve, only curves above the diagonal line joining the points (0,0) and (1,1) make sense (better than random), since below this it is interpreted as “worse than random”. The IMCP curve uses completely the domain of X and Y, i.e., the unit square. Therefore, neither the ROC and the IMCP curves, nor the areas under such curves, are comparable.

The potential of the approach and its features will be discussed in detail in the case of study.

## A case of study: tumor type prediction

To showcase the impact of imbalance on multi-class datasets, recent leading research on cancer prediction has been selected^46^. The work focus on the application of several machine learning techniques to predict the tumor tissue of origin in cases of advanced-stage metastatic tumors. This is a very important issue as a significant number of patients have no conclusive diagnoses (typically via histopathology), and they suffer from very limited therapeutic options as primary cancer type classification is a key factor in guiding treatment decisions.

The original dataset collects information from a wide range of molecular methods, and includes 4131 features extracted for classifying 35 cancer types based on driver/passenger and simple/complex mutations. A total of 6756 samples from the Hartwig Medical Foundation (metastatic tumors) and the Pan-Cancer Analysis of Whole Genomes consortium (primary tumors) were gathered. Univariate feature selection was performed to provide 463 variables, and 48 new features were generated for regional mutational density profiles by means of non-negative matrix factorization. In summary, the dataset used henceforth has 6756 samples, 511 features, and a target variable (tumor type) with 35 possible discrete values (biliary, breast, cervix, liver, pancreas, thyroid, etc.).

The multi-class nature of the dataset limits the use of some classification performance measures (e.g., the ROC curve), and its large imbalance impacts the results. Out of the 35 tumor types, the most frequent is *Breast*, with 996 samples (14.7%), and the least frequent is *Skin_Carcinoma*, with 25 samples (0.4%). The seven most frequent tumors account for more than 58%, and the seven least frequent tumors do not together reach 3.5%. In fact, 16 tumor types have less than 1% of the total, and 23 tumor types have less than they should to become a balanced dataset (2.86%).

It is important to note that the aim of this research is not to analyze the methodology proposed in^46^, but to show that, in general, good accuracy, precision, or sensitivity results for imbalanced multi-class datasets should be studied in more depth because they could lead to optimistic conclusions. In this study, the random forest (RF) technique with default parameters was chosen, as in^46^. However, isotonic regression to calibrate the probabilities produced by the RF ensemble was not necessary, as the one-versus-rest strategy to discriminate one cancer type versus other cancer types transforms the multi-class classification into multiple binary classifications, which is inappropriate for evidencing weaknesses in imbalanced multi-class classification performance measures.

Stratified 10-fold cross-validation was used to compute several classification performance measures. The accuracy, which has been shown to be class-biased, was 0.924, which would indicate that the diagnostic system is of sufficient quality. However, an analysis of the confusion matrix revealed that for 12 tumor types the precision (also class skewed) was less than 0.90 (for the tumor type *Sarcoma_Other* was 0.68), and for 13 tumor types the sensitivity was less than 0.90 (for the tumor type *Lung_SmallCell* was 0.32). In tumor prediction, which guides subsequent medical treatments, some of these low precision and sensitivity values are not acceptable.

**Figure 6:**
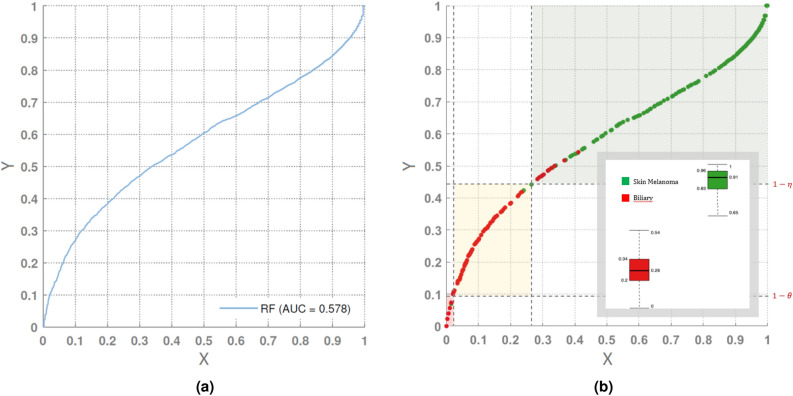
The IMCP curves for the tumor dataset.

More revealing than accuracy, precision or sensitivity, is the IMCP curve (Fig. 6a), which is based on the diagnostic system’s ability to make predictions based on the probability that the prediction is close to reality, i.e., that it does not exhibit uncertainty that has to be decided by some voting method. While a perfect diagnostic system would achieve an area under the IMCP curve of 1, the system based on RF covers only 57.8% of the ideal classification performance. The IMCP curve measures, in essence, how close these values are to 1, i.e. to assigning a cancer type with a probability close to 1. The larger the area under the IMCP curve, the lower the uncertainty of the predictive system. Therefore, what appeared to be a sound diagnostic system (accuracy $=0.924$) may not be so reliable (AU(IMCP)$=0.578$).

The incorrect ($\theta$) and correct ($\eta$) classification thresholds provide the landscape of certainty for the dataset: [0, 0.088) for incorrect classifications (72 samples: 1.06%), [0.088, 0.459] for uncertainty (1023 samples: 15.14%), and (0.459, 1] for correct classifications (5661 samples: 83.79%). These values, related to the percentage of samples, are very illustrative because are delimiting the proportion of samples that could be correctly classified ($[83.79\%,98.93\%]$). However, confidence in prediction can vary substantially. This fact is of extreme importance in some predictive systems, such as in the case of tumor diagnosis.

The IMCP curve also allows to analyze in depth the behavior of the classifier for each class, which is very useful for experts, as they could re-evaluate the prediction provided by the system, and possibly supplement it with other factors in case of low reliability. To explain the nature of the predictions, Fig. 6b illustrates the best (*Skin_Melanoma*) and the worst (*Biliary*) classification performances in a graph showing all samples for only both classes, in green and red, respectively (the same values displayed in the IMCP curve in Fig. 6a). Most of the predictions for *Skin_Melanoma* are above $1-\eta$ within the green area (correct classification). However, most of the predictions for *Biliary* are between $1-\theta$ and $1-\eta$, within the yellow area (uncertainty). This means that most of the predictions were unreliable, in the sense that the classifier could assign to other tumor types prediction probabilities lower but very close to the predicted tumor.

The values of $\varphi ^\prime$ for each class are also depicted in box-plots, illustrating that the medians are 0.26 (*Biliary*) and 0.91 (*Skin_Melanoma*). The Q3 value for *Biliary* (0.34) indicates that 75% of the predictions are extremely low. In contrast, the Q1 value for *Skin_Melanoma* (0.83) indicates that 75% of the predictions are excellent. In summary, the reliability of the diagnostic system is not similar for all the tumor types. The expert may have high confidence in a prediction for *Skin_Melanoma*, but very low confidence in a prediction of *Biliary*.

**Figure 7:**
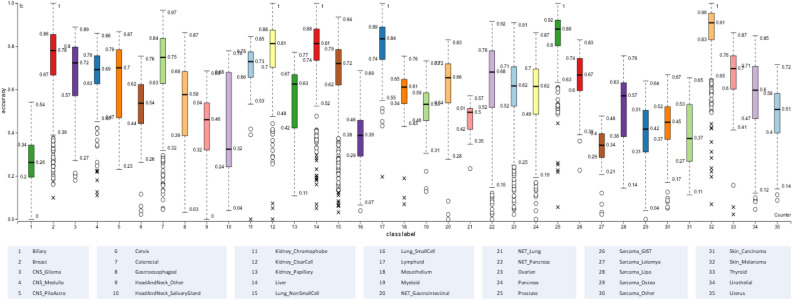
Conditional classification performance for each tumor type.

In total, 7 out of 37 tumor types had medians below $1-\eta$ (more than 50% of samples under the correct classification threshold), and only 19 out of 35 had the Q1 value above $1-\eta$ (more than 75% of samples over the correct classification threshold). An overall conditional classification performance approach is depicted in Fig. 7, in which it is easy to detect the diagnostic ability of the system for each type of tumor. The information provided by the figure is very valuable from a diagnostic system quality perspective, as it not only highlights the tumor types for which the system is reliable but also identifies the tumor types for which predictions should be examined more carefully by experts in the field. In addition, the conditional classification performance results identify to which classes greater efforts should be applied, from a data analytics perspective, to improve the quality of predictions.

In summary, a thorough analysis of the quality of the diagnostic system, in principle with excellent indicators (accuracy = 0.924, average precision = 0.908, average sensitivity = 0.866, mean F_1_-score = 0.877), reveals that these measures are very sensitive to imbalance, as the classification performance provided by the area under the IMCP curve is 0.578, mainly due to low confidence on predictions for some tumor types.

The analysis performed with this dataset is extensible to any dataset. It extracts interesting aspects about the overall quality of the diagnostic system and also about the classification performance for each class. The method is useful for multi-class datasets, and especially suitable for more than two classes (where the ROC curve is not applicable). Furthermore, it is insensitive to class imbalance, which adds further benefit to the examination of the performance of predictive systems.

## Conclusion

Classification models are designed to predict unseen events from what has been learned during training. The quality of the model indicates how good the prediction would be in the future, and can be measured in different ways. Several important performance measures are not valid for multi-class datasets (e.g., the ROC curve), and many of them fail at analyzing imbalanced datasets (e.g., the accuracy or the F_β_-score). The aim of this work is to provide a novel method, named the IMCP curve, that graphically represents the classification performance for both multi-class and imbalanced datasets. The usefulness is broad, particularly in biomedicine, as most of the gene expression datasets are imbalanced (e.g., proportion of cancerous versus healthy samples) and many of them include more than two target-categories (e.g., subtypes of tumors).

The IMCP reveals that some quality measures could be optimistic, as they provide overall values of classification performance of the diagnostic system without taking into account the class bias. Moreover, the approach facilitates an in-depth conditional analysis of the diagnostic system, firstly by identifying which classes perform better (high confidence), and secondly by allowing further research on specific target classes that perform worse (high risk).

The promising scenario opened by the IMCP curve requires further analysis and study of the properties in relation to the most commonly used metrics in the scientific literature, since prediction probabilities have much more potential than the confusion matrix.

### Supplementary Information

Supplementary Information.

## Data Availability

The datasets used in the “Rationale” section are publicly available at https://github.com/adaa-polsl/imcp/tree/main/tests with the following names: Dataset A: A$\_$exp-0.base-100.csv Dataset B: B$\_$exp-2.base-100.csv Dataset C: C$\_$exp-4.base-100.csv Dataset D: D$\_$exp-6.base-100.csv. A “Case study” data are taken from: Nguyen et al.^46^. This dataset is public, as indicated in the “Data availability” section of the aforementioned reference and is freely accessible for academic purposes (Supplementary Information). To generate the IMCP curves, all experiments used the Python package publicly available at https://github.com/adaa-polsl/imcp.
