# Supplementary material for: Classification performance assessment for imbalanced multiclass data
Source: Sci Rep. 2024 May 10;14:10759. doi: 10.1038/s41598-024-61365-z (PMC11087593; doi:10.1038/s41598-024-61365-z)
Supplement: Supplementary file 1 — Supplementary Information. [file 41598_2024_61365_MOESM1_ESM.pdf]

In this work, two experiments have been conducted using different datasets, which are described below.

Section: Rationale

An analysis is performed on how class imbalance affects classification performance, quantified using the accuracy measure. To achieve this, starting from an identical distribution function for two variables with three classes, four datasets have been generated, identified in the article as datasets A, B, C, and D. Each of them varies the imbalance in the proportions as indicated in Figure 2. The goal of this experimentation is to demonstrate that as the imbalance increases, accuracy improves, both when using Naive-Bayes and Random Forests. However, when using the IMCP measure, it can be observed from Figure 3 that the area under the IMCP curve worsens as the imbalance increases, a circumstance that appears much more natural.

Section: A Case Study: Tumor Type Prediction

An analysis is carried out to showcase the potential of the IMCP measure with respect to a real dataset characterized by class imbalance and multiclass classification (comprising 35 tumor types). The dataset is extensively described in our article and is also detailed in the original paper where this dataset was first introduced: Nguyen, L., Van Hoeck, A. & Cuppen, E. Machine learning–based tissue of origin classification for cancer of unknown primary diagnostics using genome–wide mutation features. Nat. Commun. 13, 4013 (2022). https://doi.org/10.1038/s41467-022-31666-w
